# Clonality, virulence determinants, and profiles of resistance of clinical *Acinetobacter baumannii* isolates obtained from a Spanish hospital

**DOI:** 10.1371/journal.pone.0176824

**Published:** 2017-04-27

**Authors:** Elias Dahdouh, Rosa Gómez-Gil, Sonsoles Pacho, Jesús Mingorance, Ziad Daoud, Monica Suárez

**Affiliations:** 1 Department of Animal Health, Faculty of Veterinary, Universidad Complutense de Madrid, Madrid, Spain; 2 Servicio de Microbiología, IdiPAZ, Hospital Universitario La Paz, Madrid, Spain; 3 Department of Clinical Microbiology, Faculty of Medicine, University of Balamand, Balamand, Lebanon; Leibniz-Institute DSMZ, GERMANY

## Abstract

**Introduction:**

*Acinetobacter baumannii* is a nosocomial pathogen that is showing increasing rates of carbapenem resistance. Multi-Drug Resistant (MDR) International Clones (ICs), associated with certain oxacillinases, are being reported globally. This organism also harbors numerous virulence determinants. In this study, we aim at characterizing *A*. *baumannii* isolated from a Spanish hospital in terms of antimicrobial susceptibility, clonality, carbapenemase genes harbored, and virulence determinants expressed.

**Materials and methods:**

Fifty nine clinical bloodstream isolates were obtained from 2009 until 2013. Antimicrobial Susceptibility Testing was performed according to the CLSI guidelines. PFGE and tri-locus PCR typing were then performed in order to determine local and international clonality. PCRs for the detection of common carbapenemases were also performed. Production of hemolysis, biofilms, siderophores, surface motility, and proteolysis were determined phenotypically. Doubling times for selected strains were also calculated. Finally, statistical analysis for detecting associations between these factors was conducted.

**Results and discussion:**

Carbapenem non-susceptibility was 84.75%, suggesting the immediate need for intervention. PFGE showed the distribution of the majority of the isolates among 7 clusters. Although all three ICs were detected, IC II was predominant at 71.19%. *bla*_OXA-24-like_ was the most prevalent carbapenemase (62.71%), followed by *bla*_OXA-58-like_ (13.56%), and *bla*_OXA-23-like_ (11.86%). Strains pertaining to IC II, and those harboring *bla*_OXA-24-like_, were positively associated with α-hemolysis, production of strong biofilms, and siderophore production. Harboring *bla*_OXA-23-like_ and *bla*_OXA-58-like_ was associated with attenuated virulence. These associations suggest that an interplay exists between these factors that could be locally exploited.

**Conclusions:**

An alarmingly high rate of carbapenem non-susceptibility has been detected in this study. There was a predominance of IC II and *bla*_OXA-24-like_, and those factors were associated with heightened expression of virulence determinants. This association could be exploited for modifying treatment regimens and for improving on infection control protocols in this hospital.

## Introduction

*Acinetobacter baumannii* is an opportunistic nosocomial pathogen that has caused severe outbreaks all around the world [1]. Secondary infections with *A*. *baumannii* could cause ventilator-associated pneumonia, burn wound infections, prosthetic-related infections, and bacteremia [2]. Infections with Multi-Drug Resistant (MDR) *A*. *baumannii* isolates and improper empirical treatments increase mortality rates among critically ill patients [3]. In addition to the wide array of intrinsic mechanisms of antimicrobial resistance found in *A*. *baumannii*, this bacterium has a heightened ability of acquiring resistance [4].

Carbapenems have long been the treatment of choice for MDR *A*. *baumannii*. However, rates of carbapenem resistance are increasing worldwide [5]. Mortality rates associated with Carbapenem Resistant *Acinetobacter baumannii* (CRAB) isolates could reach as high as 33% [6]. Numerous mechanisms of carbapenem resistance were identified in *A*. *baumannii*. Of these, Oxacillinases (OXAs), mainly OXA-23-like, OXA-24-like, and OXA-58-like, are the most common cause of resistance to these antimicrobial agents [7]. Moreover, a few globally disseminated International Clones (ICs), that include IC I, IC II, and IC III, were found to be the most successful clones that cause outbreaks on a global scale [8]. CRAB isolates pertaining to all three ICs are being reported from all around to world [9, 10]. In Spain, a notoriously high incidence of CRAB isolates, with a predominance of IC II, is found [11, 12]. One study showed that imipenem resistance among *A*. *baumannii* isolates from Spain increased from 74.2% in 2000 to 89.2% in 2010 [13]. A CRAB incidence rate of 43% among patients in Spanish Intensive Care Units (ICUs) was reported from another study [14]. Moreover, although *bla*_OXA-23_ was reported in some strains [15], *bla*_OXA-24_ was found to be the predominant OXA in Spain [16].

The pathogenicity of *A*. *baumannii* is not yet fully understood. However, several factors have been associated with its virulence [17]. One such factor is OmpA which is able to bind to epithelial cells and induce eukaryotic cell death [18]. Moreover, CsuE, which is part of a chaperon-usher pili assembly system, is involved in biofilm formation in this organism [19]. Additionally, despite its classification as a non-motile organism, certain *A*. *baumannii* strains have shown twitching surface motility [20]. Hemolysis on Sheep Blood Agar (SBA), exoprotease activity, and siderophore production were also reported among *A*. *baumannii* strains [21]. A combination of methods have been used to demonstrate that the deletion or disruption of the respective genes coding for the aforementioned factors did indeed result in reduced *in-vivo* virulence of *A*. *baumannii* [22, 23].

A complex relationship seems to exist between virulence and antimicrobial resistance. One study demonstrated reduced virulence after the acquisition of carbapenem resistance through non-enzymatic means [24]. Another study showed that the acquisition of the PER-1 beta-lactamase actually resulted in increased virulence [25]. Nevertheless, despite the great importance of OXAs in clinical *A*. *baumannii* isolates, the effect of these enzymes on virulence is not vigorously investigated [26]. A study recently performed by part of our research group investigated this relationship among a set of *A*. *baumannii* isolates obtained from Lebanese patients. This study concluded that there was no relationship between virulence determinants and resistance in this set of isolates, but among certain virulence determinants in particular [27]. However, one limitation of that study was that the isolates obtained had very low diversity, which could possibly obscure some associations between the tested factors.

In this study, we aim to characterize *A*. *baumannii* nosocomial isolates obtained from Spanish patients in terms of antimicrobial resistance, virulence determinants, prevalence of carbapenemases, and clonal relatedness. We also aim at investigating any association between these factors that could have an impact on the treatment of CRAB infections. We found that high rates of carbapenem resistance are present among the collected isolates and that only a handful of clones were responsible for causing repeated infections over a five-year period. Additionally, we found that international clone II and OXA-24-like were associated with increased expression of virulence determinants as compared to the other clones and oxacillinases. This association could be locally exploited by healthcare professionals in order to improve on treatment outcomes and infection control protocols. Moreover, our findings show that an interplay could exist between certain mechanisms of resistance and the expression of virulence determinants.

## Results

### Distribution of bacterial isolates

A total of 59 consecutive non-repetitive bloodstream *A*. *baumannii* isolates were obtained from Hospital Universitario–La Paz (HU-LP) located in Madrid, Spain. Thirty (50.85%) of the isolates were collected in 2009, nine (15.25%) in 2010, five (8.47%) in 2011, eleven (18.65%) in 2012, and four (6.78%) in 2013. Twenty six (44.07%) of these isolates were obtained from the ICU, 22 (37.29%) from the Critical Care Nursing Unit (CCNU), two (3.39%) from each of the Emergency (EM), Resuscitation (RE), and Internal Medicine (IM) wards, and one (1.69%) from each of the Nephrology (NE), Hematology/Oncology (HO), Cardio/Thoracic (CT), General Surgery (GS), and Neonatal (NEO) wards.

### Antibiotic susceptibility profiles

**Fig 1:**
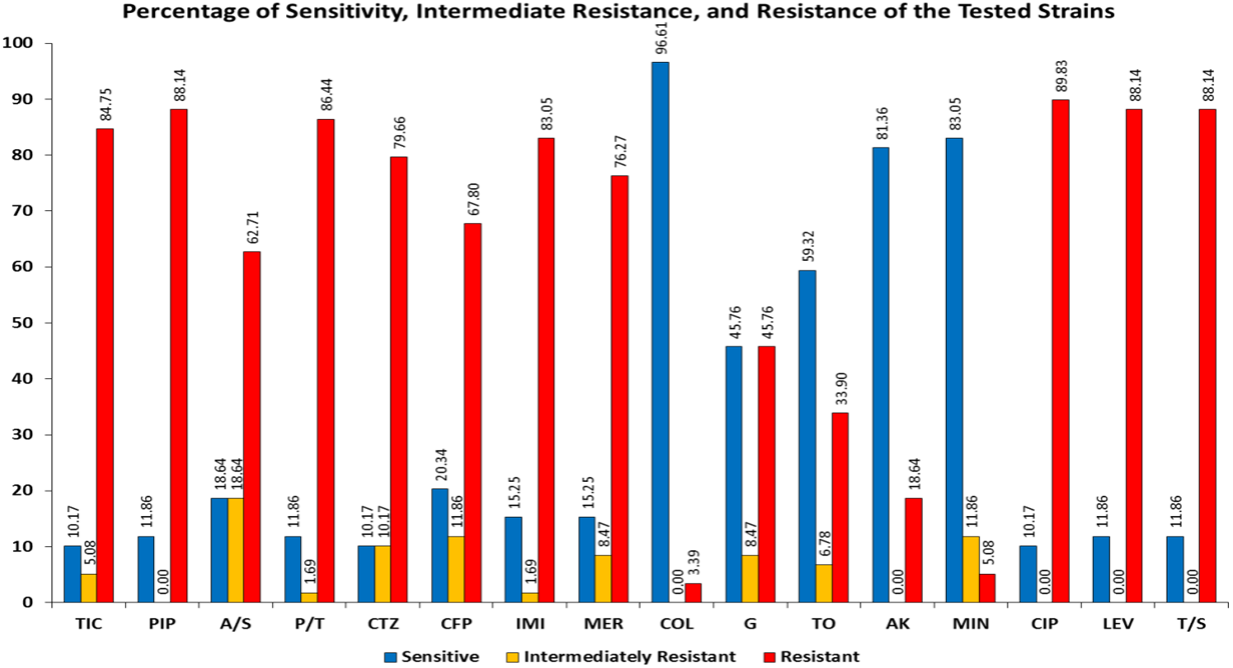
Percentage of sensitive (blue), intermediately resistant (yellow), and resistant (red) isolates to the different antimicrobial agents. “TIC” stands for ticarcillin, “PIP” for piperacillin, “A/S” for ampicillin/sulbactam, “P/T” for piperacillin/tazobactam, “CTZ” for ceftazidime, “CFP” for cefepime, “IMI” for imipenem, “MER” for meropenem, “COL” for colistin, “G” for gentamicin, “TO” for tobramycin, “AK” for amikacin, “MIN” for minocycline, “CIP” for ciprofloxacin, “LEV” levofloxacin, and “T/S” for trimethoprim/sulfamethoxazole.

Antibiotic susceptibility profiles were obtained by determining the Minimum Inhibitory Concentration (MIC) values and interpreting them according to the CLSI guidelines [28]. Fig 1 shows the percentage of sensitivity, intermediate resistance, and resistance of the tested *A*. *baumannii* isolates. The MIC values are found in S1 Table. More than 80% of the isolates were non-sensitive to ticarcillin, piperacillin, ampicillin/sulbactam, piperacillin/tazobactam, ceftazidime, ciprofloxacin, levofloxacin, and trimethoprim/sulfamethoxazole. 84.75% of the isolates were non-susceptible to carbapenems, 79.66% to cefepime and 16.95% to minocycline. Non-susceptibility to aminoglycosides ranged from 18.64% for amikacin to 54.24% for gentamycin. Only two isolates (3.39%) were resistant to colistin.

### Detection of carbapenemases and virulence genes

PCRs for common carbapenemases, in addition to certain genes involved in virulence were performed. The intrinsic *bla*_OXA-51-like_ gene was detected in 56 (94.92%) of the isolates whereas *bla*_OXA-48_, *bla*_NDM_, and *bla*_KPC_ were not detected in any isolate. *bla*_OXA-24-like_ was the most prevalent carbapenemase gene where it was detected in 37 (62.71%) isolates, followed by *bla*_OXA-58-like_ in 8 (13.56%) isolates, and *bla*_OXA-23-like_ in 7 (11.86%) isolates. Isolate 50 harbored both *bla*_OXA-24-like_ and *bla*_OXA-58-like_. All the isolates were positive for *ompA* while 57 strains (96.61%) were positive for *csuE*.

### Clusters, clones and carbapenemases

**Fig 2:**
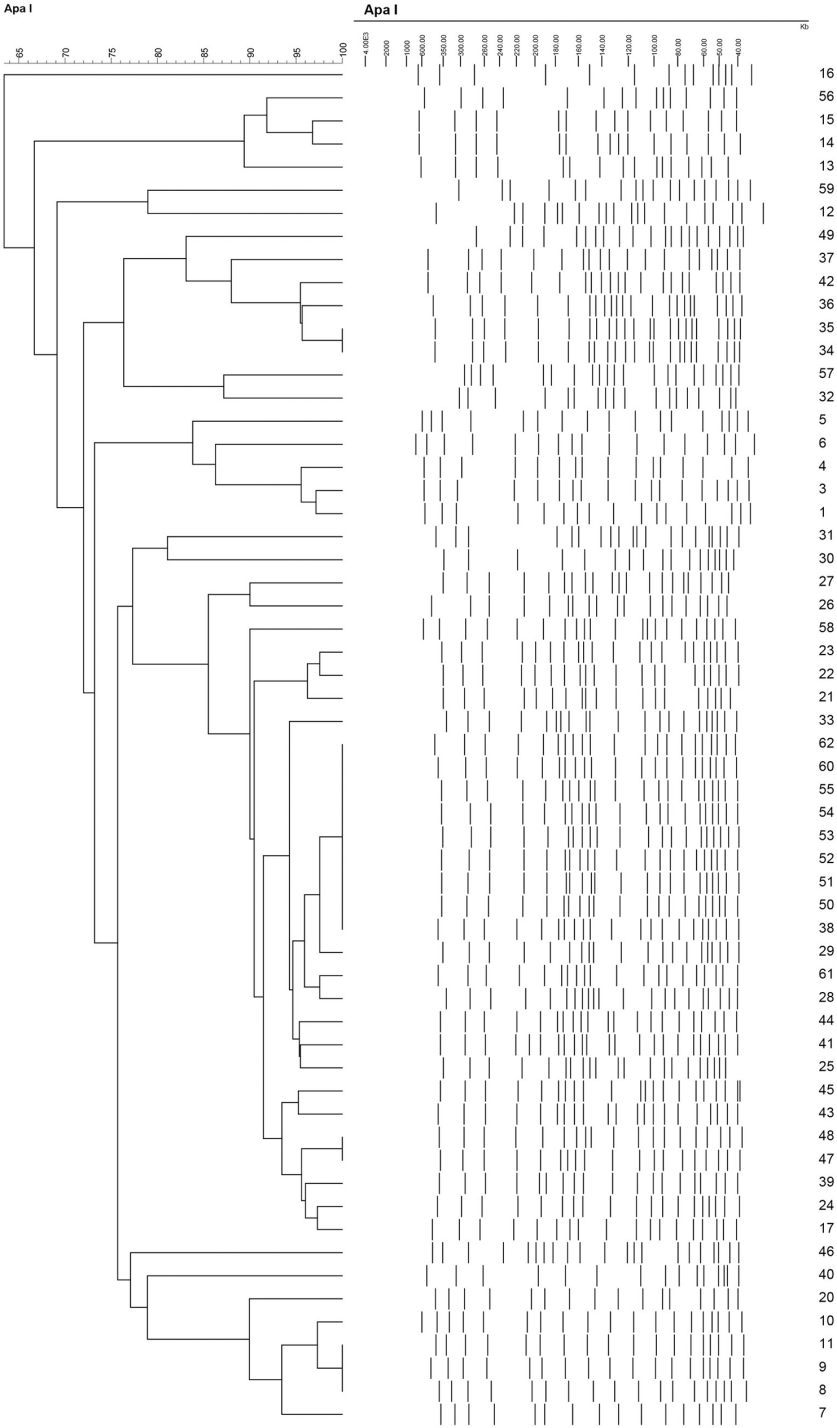
The dendrogram constructed from the PFGE analysis using the Unweighted Pair Group Method with Arithmetic Mean (UPGMA) with 1.5% tolerance and 1.5% optimization.

Pulsed Field Gel Electrophoresis (PFGE) was performed in order to determine the local clonality of the isolates (Fig 2). Tri-locus multiplex PCRs were then performed in order to determine the global lineage of the strains. Table 1 shows the isolation ward, isolation date, detected carbapenemases, as well as the ICs that the isolates pertained to. Five isolates did not belong to any cluster while the rest of the isolates were spread across seven distinct clusters. All the isolates were found to pertain to a particular IC or group according to the scheme summarized by Karah *et al*. [8], except for seven. Of these seven isolates, *bla*_OXA-66_ was amplified from the first multiplex and *ompA* from the second for isolates 12 and 13; o*mpA* from multiplex 1 and *bla*_OXA-69_ from multiplex 2 for isolate 57; only c*suE* from multiplex 1 for isolate 59; and, despite several attempts, no genes were amplified from any multiplex PCR for isolates 16, 32, and 46. Of the isolates that did not pertain to any IC, only isolates 13, 57, and 32 pertained to a particular cluster by PFGE (Table 1).

**Table 1:** Results of the dendrogram obtained from PFGE, in addition to which International Clone the tested isolated belonged to, which ward they were isolated from, the date of isolation, susceptibility to carbapenems, the detected carbapenemases, and some virulence determinants.

	Epidemiological Data			Resistance					Virulence		
Strain	Cluster	Clone	Ward	Isolation Date	Carb. R	OXA-23	OXA-24	OXA-58	Hemolysis	Biofilm	Siderophores
16	-	-	NE	17/12/2012	-	-	-	-	-	++	+
56	C1	IC III	CCNU	10/6/2009	+	-	+	-	-	+	+
15	C1	IC III	ICU	12/11/2012	+	-	+	-	-	+	+
14	C1	IC III	CCNU	10/10/2012	+	-	+	-	-	+	+
13	C1	-	ICU	26/9/2012	+	-	+	-	+	+	+
59	-	-	IM	18/5/2010	-	-	-	-	+	++	+
12	-	-	CCNU	20/8/2012	+	-	+	-	+	++	-
49	C2	IC II	ICU	1/10/2009	+	-	+	-	+	+	+
37	C2	IC II	CCNU	19/9/2009	+	-	+	-	+	++	+
42	C2	IC I	CCNU	15/12/2009	+	-	-	+	-	+	-
36	C2	IC I	CCNU	25/9/2009	+	-	-	+	-	+	-
35	C2	IC I	CCNU	6/10/2009	+	-	-	+	-	++	-
34	C2	IC I	CCNU	7/12/2009	+	-	-	+	-	++	-
57	C3	-	ICU	25/11/2009	-	-	-	-	-	++	+
32	C3	-	CT	24/9/2009	-	-	-	-	-	++	-
5	C4	IC II	ICU	21/12/2011	+	+	-	-	-	++	+
6	C4	IC II	CCNU	29/12/2011	+	-	+	-	+	++	-
4	C4	IC II	ICU	10/1/2012	+	-	+	-	+	++	-
3	C4	IC II	CCNU	22/6/2011	+	-	+	-	+	++	+
1	C4	IC II	CCNU	12/1/2011	+	-	+	-	+	++	-
31	C5	G 14	CCNU	29/12/2009	-	-	-	-	-	++	+
30	C5	IC II	HO	14/4/2009	-	-	-	-	-	++	+
27	C6	IC II	ICU	25/5/2009	+	-	+	-	+	++	-
26	C6	IC II	ICU	4/5/2010	-	-	-	-	+	++	+
58	C6	IC III	CCNU	24/6/2009	+	-	+	-	-	++	-
23	C6	IC II	CCNU	22/3/2013	+	-	+	-	-	++	-
22	C6	IC II	CCNU	20/3/2013	+	-	+	-	-	++	+
21	C6	IC II	EM	4/2/2013	+	-	+	-	-	++	+
33	C6	IC II	RE	31/3/2009	+	-	+	-	+	++	+
62	C6	IC II	ICU	9/1/2009	+	-	+	-	+	++	+
60	C6	IC II	ICU	28/4/2010	+	-	-	-	+	++	-
55	C6	IC II	ICU	14/1/2009	+	-	+	-	+	++	+
54	C6	IC II	ICU	9/1/2009	+	-	+	-	+	++	-
53	C6	IC II	EM	3/11/2009	I	-	+	-	+	++	+
52	C6	IC II	ICU	21/1/2010	+	-	+	-	+	++	+
51	C6	IC II	ICU	16/12/2009	+	-	+	-	+	++	+
50	C6	IC II	ICU	27/8/2009	+	-	+	+	+	++	+
38	C6	IC II	ICU	3/11/2009	-	-	-	-	-	++	-
29	C6	IC II	RE	16/2/2009	+	-	+	-	-	++	+
61	C6	IC II	ICU	7/1/2009	+	-	+	-	+	++	+
28	C6	IC II	ICU	25/2/2010	+	-	+	-	+	++	+
44	C6	IC II	IM	13/10/2009	+	-	+	-	+	++	+
41	C6	IC I	CCNU	9/12/2009	+	-	-	+	-	+	-
25	C6	IC II	CCNU	23/6/2009	+	-	+	-	+	++	+
45	C6	IC II	CCNU	27/1/2010	+	-	-	+	+	++	+
43	C6	IC II	ICU	21/5/2009	+	-	+	-	+	++	+
48	C6	IC II	ICU	9/6/2010	+	-	+	-	+	++	+
47	C6	IC II	ICU	28/9/2010	+	-	+	-	+	++	+
39	C6	IC II	GS	9/2/2010	+	-	+	-	+	++	+
24	C6	IC II	CCNU	16/9/2009	+	-	+	-	+	++	+
17	C6	IC II	ICU	20/11/2012	+	-	+	-	+	++	+
46	-	-	NEO	9/9/2009	-	-	-	-	-	++	-
40	-	IC II	ICU	6/11/2009	+	-	+	-	+	++	+
20	C7	IC II	CCNU	9/1/2013	+	+	-	-	-	++	-
10	C7	IC II	CCNU	8/5/2012	+	+	-	-	-	++	+
11	C7	IC II	ICU	21/12/2011	+	+	-	-	-	++	+
9	C7	IC II	CCNU	17/4/2012	+	+	-	-	-	++	+
8	C7	IC II	ICU	15/3/2012	+	+	-	-	-	++	+
7	C7	IC II	ICU	10/1/2012	+	+	-	-	-	++	+

“Carb R.” stands for carbapenem resistance, “C1 till C7” stand for Cluster 1 to Cluster 7, “IC” stands for international clone, “G” stands for group, and “I” stands for intermediate resistance. The wards are as follows: “CCNU” is the Critical Care Nursing Unit, “ICU” is the Intensive Care Unit, “EM” is Emergencies, “RE” is Resuscitation, “IM” is Internal Medicine, “NE” is Nephrology, “HO” is Hematology/Oncology, “CT” is Cardio/Thoracic, “GS” is General Surgery, and “NEO” is Neonatal.

Three out of four isolates from Cluster 1 (C1) pertained to IC III and harbored *bla*_OXA-24-like_. Two of the isolates from Cluster 2 (C2) had a slightly different pulsotype from the other four in that cluster, pertained to IC II, and had *bla*_OXA-24-like_. The other four isolates in C2 pertained to IC I and harbored the *bla*_OXA-58-like_ gene. All the isolates from this cluster were isolated in 2009 and none was detected in subsequent years. Cluster 3 (C3) consists of two carbapenem sensitive isolates that did not pertain to any IC. Four out of five of the isolates of Cluster 4 (C4) pertained to IC II and were resistant to carbapenems. The fifth isolate had a slightly different pulsotype and harbored *bla*_OXA-23-like_ instead of the *bla*_OXA-24-like_ gene harbored by the others in this cluster. Cluster 5 (C5) included two carbapenem sensitive isolates with one pertaining to IC II and the other to a less disseminated clone designated as Group 14 [8].

The largest cluster identified, containing 29 isolates, was Cluster 6 (C6). All the isolates in this cluster were carbapenem resistant, except for isolate 53 which was intermediately resistant, and isolates 26 and 38 which were sensitive. All of the carbapenem non-sensitive clones of C6 harbored *bla*_OXA-24-like_, with the exception of isolates 41 and 45 that harbored *bla*_OXA-58-like_ instead, and isolate 60 which harbored none of the tested carbapenemase genes. In addition to *bla*_OXA-24-like_, isolate 50 also harbored *bla*_OXA-58-like_. The majority of isolates were obtained from the ICU but this clone was also detected in several other hospital wards (Table 1). Moreover, isolates pertaining to this cluster were detected in 2009, 2010, 2012, and 2013, demonstrating the persistence of this clone. All the isolates from this cluster belonged to IC II, except for isolate 58 which belonged to IC III and 41 which belonged to IC I.

The isolates of Cluster 7 (C7) harbored *bla*_OXA-23-like_, belonged to IC II, and were detected in 2011, 2012, and 2013. Of the five isolates that did not belong to any cluster, only isolate 40 pertained to IC II whereas the rest did not belong to any IC. This isolate had 79% similarity with C7 but was not considered as part of the cluster because the cutoff was set at 80%. Isolates 12 and 40 were resistant to carbapenems and harbored the *bla*_OXA-24-like_ gene. Additionally, isolate 12 was resistant to colistin. In total, 5 (8.47%) isolates pertained to IC I, 42 (71.19%) to IC II, 4 (6.78%) to IC III, 8 (13.56%) did not belong to any IC, and one to Group 14.

### Determination of the virulence factors, and their association with clonality and carbapenemases

Virulence determinants were phenotypically tested for in all isolates, and representative isolates were selected for the determination of doubling times (see Materials and Methods). The obtained data was then statistically analyzed in order to detect any associations between the factors that were tested for. Table 2 shows the results of biofilm formation, hemolysis, siderophore production, and proteolytic activity. Additionally, ICs, PFGE clusters, and doubling times for selected isolates are shown in that table. No statistical association was detected when the virulence factors were compared one to another. However, IC I was negatively associated with α-hemolysis on SBA, siderophore production, and strong biofilm formation (p<0.05). IC II on the other hand was positively associated with these three factors (p<0.05). Nevertheless, all the isolates of C7, which pertained to IC II but had *bla*_OXA-23-like_, were negative for α-hemolysis on SBA. IC III was negatively associated with α-hemolysis on Sheep Blood Agar (SBA) and production of strong biofilms (p<0.05).

**Table 2:** Results of the virulence factors experiments for the tested strains, in addition to which cluster and international clone they belong to.

Strain	Cluster	Clone	Hemolysis	Biofilm	Siderophores	Proteolytic Activity (U/L)	Doubling Time (hours)
16	-	-	-	++	+	25.92 ± 3.10	ND
56	C1	IC III	-	+	+	20.85 ± 8.01	0.386 ± 0.041
15	C1	IC III	-	+	+	30.09 ± 1.86	0.559 ± 0.127
14	C1	IC III	-	+	+	25.92 ± 1.79	ND
13	C1	-	α - D3	+	+	9.24 ± 2.06	0.461 ± 0.031
59	-	-	α - D2	++	+	35.15 ± 3.14	0.519 ± 0.079
12	-	-	α - D3	++	-	12.51 ± 3.58	0.666 ± 0.037
49	C2	IC II	α - D2	+	+	23.54 ± 7.81	0.401 ± 0.023
37	C2	IC II	α - D2	++	+	23.24 ± 5.44	0.382 ± 0.018
42	C2	IC I	-	+	-	35.45 ± 3.14	ND
36	C2	IC I	-	+	-	19.96 ± 2.87	ND
35	C2	IC I	-	++	-	17.88 ± 3.22	0.613 ± 0.082
34	C2	IC I	-	++	-	14.60 ± 6.34	ND
57	C3	-	-	++	+	48.26 ± 11.41	ND
32	C3	-	-	++	-	17.88 ± 1.55	0.577 ± 0.053
5	C4	IC II	-	++	+	27.71 ± 3.10	0.436 ± 0.040
6	C4	IC II	α - D1	++	-	29.20 ± 10.28	ND
4	C4	IC II	α - D2	++	-	34.26 ± 2.58	ND
3	C4	IC II	α - D2	++	+	26.81 ± 2.36	0.449 ± 0.042
1	C4	IC II	α - D2	++	-	39.62 ± 8.68	ND
31	C5	G 14	-	++	+	12.21 ± 5.08	ND
30	C5	IC II	-	++	+	28.60 ± 7.09	0.384 ± 0.038
27	C6	IC II	α - D1	++	-	27.41 ± 2.87	ND
26	C6	IC II	α - D2	++	+	35.15 ± 4.41	0.342 ± 0.077
58	C6	IC III	-	++	-	30.39 ± 9.67	ND
23	C6	IC II	-	++	-	22.34 ± 3.90	ND
22	C6	IC II	-	++	+	31.28 ± 1.55	0.340 ± 0.069
21	C6	IC II	-	++	+	34.86 ± 2.36	ND
33	C6	IC II	α - D1	++	+	14.30 ± 3.90	ND
62	C6	IC II	α - D2	++	+	9.24 ± 1.37	ND
60	C6	IC II	α - D2	++	-	17.88 ± 0.89	ND
55	C6	IC II	α - D1	++	+	31.58 ± 19.18	ND
54	C6	IC II	α - D2	++	-	24.73 ± 3.14	ND
53	C6	IC II	α - D1	++	+	25.03 ± 5.58	0.428 ± 0.017
52	C6	IC II	α - D2	++	+	24.13 ± 4.98	0.436 ± 0.088
51	C6	IC II	α - D2	++	+	27.41 ± 3.38	ND
50	C6	IC II	α - D2	++	+	24.43 ± 2.58	ND
38	C6	IC II	-	++	-	28.90 ± 4.03	0.482 ± 0.049
29	C6	IC II	-	++	+	23.24 ± 0.89	ND
61	C6	IC II	α - D2	++	+	13.70 ± 7.22	ND
28	C6	IC II	α - D2	++	+	24.73 ± 3.14	ND
44	C6	IC II	α - D2	++	+	29.49 ± 4.47	ND
41	C6	IC I	-	+	-	18.47 ± 1.37	0.651 ± 0.026
25	C6	IC II	α - D1	++	+	28.00 ± 2.73	ND
45	C6	IC II	α - D2	++	+	42.90 ± 7.74	0.324 ± 0.027
43	C6	IC II	α - D2	++	+	31.88 ± 10.66	ND
48	C6	IC II	α - D2	++	+	21.15 ± 1.86	ND
47	C6	IC II	α - D2	++	+	25.03 ± 4.64	ND
39	C6	IC II	α - D2	++	+	29.20 ± 7.71	ND
24	C6	IC II	α - D1	++	+	33.37 ± 8.30	ND
17	C6	IC II	α - D1	++	+	23.54 ± 2.06	0.462 ± 0.016
46	-	-	-	++	-	24.73 ± 4.22	ND
40	-	IC II	α - D2	++	+	47.67 ± 13.68	ND
20	C7	IC II	-	++	-	23.24 ± 3.10	0.350 ± 0.033
10	C7	IC II	-	++	+	28.00 ± 7.60	ND
11	C7	IC II	-	++	+	29.49 ± 6.26	ND
9	C7	IC II	-	++	+	29.20 ± 7.60	0.379 ± 0.019
8	C7	IC II	-	++	+	36.35 ± 7.22	ND
7	C7	IC II	-	++	+	37.84 ± 1.86	ND

“C1 till C7” stand for Cluster 1 to Cluster 7, “IC” stands for international clone, “G” stands for group, and “ND” stands for not determined. In the column of hemolysis, “α” designates the type of hemolysis detected and “D1 to D3” designates the day in which hemolysis was first detected. The averages and standard deviations presented in the last two columns are a result of 3 independent experiments.

In terms of carbapenemase genes, harboring *bla*_OXA-23-like_ was negatively associated with α-hemolysis on SBA while harboring *bla*_OXA-24-like_ was positively associated with α-hemolysis and siderophore production (p<0.05). Harboring *bla*_OXA-58-like_ was negatively associated with siderophore production and α-hemolysis (p<0.05). Proteolytic activity was not associated with any of the other factors (p<0.05). The highest level of proteolytic activity (48.26 ± 11.41 U/L) was detected for the carbapenem-sensitive isolate 57. This isolate was negative for α-hemolysis on SBA, a strong biofilm former, produced siderophores, and had the highest motility diameter (8.92 ± 4.97 mm). The carbapenem resistant, *bla*_OXA-24-like_ harboring, α-hemolytic, siderophore producing, isolate 40 also had a high proteolytic activity (47.67 ± 13.68 U/L). However, it showed no surface motility. Isolates 13 and 62 showed the lowest proteolytic activity (9.24 ± 2.06 and 9.24 ± 1.37 U/L, respectively). Both were α-hemolytic, showed no surface motility, were resistant to carbapenems, harbored *bla*_OXA-24-like_, and produced siderophores. The only difference between them was that isolate 13 was a weak biofilm former whereas isolate 62 was a strong biofilm former.

In terms of motility, only isolates 20, 25, 26, 27, 31, and 57 showed a circular diffusion pattern on 0.3% LB-Agar, indicative of surface motility. The motility diameters ranged from 33.33 ± 4.92 mm for isolate 57 to 8.17 ± 1.93 for isolate 31. Isolates 20, 25, 26, 27, and 31 had very similar diffusion diameters one to the other. The only common factor found among these isolates was that they were all strong biofilm formers and that the two that were selected for the calculation of doubling times (20 and 26) showed a relatively fast doubling time (0.342 ± 0.077 and 0.350 ± 0.033 hours, respectively). The slowest doubling time was detected for isolate 12 (0.666 ± 0.037 hours), which was resistant to colistin and carbapenems, and harbored *bla*_OXA-24-like_. This isolate showed α-hemolysis on SBA and was a strong biofilm former, but was negative for siderophore production. The two closest isolates to it were isolates 41 and 35 which showed doubling times of 0.651 ± 0.026 and 0.613 ± 0.082 hours, respectively. Both harbored the *bla*_OXA-58-like_ gene and were negative for α-hemolysis on SBA and siderophore production. Isolate 35 was a strong biofilm former whereas isolate 41 was a weak biofilm former. The fastest doubling time was detected for the other colistin resistant isolate, isolate 45 (0.324 ± 0.027 hours). Interestingly, this isolate harbored *bla*_OXA-58-like_, in addition to being a strong biofilm former, being positive for α-hemolysis on SBA and siderophore production, and having a high proteolytic activity (42.9 ± 7.74 U/L).

## Discussion

This study describes the phenotypic and genotypic characteristics of 59 *A*. *baumannii* isolates collected over five years from a Spanish hospital. It shows the predominance of the MDR IC II in this hospital and its prolonged persistence, which falls in agreement with national and international epidemiological data [8, 12]. Nevertheless, the method of comparison used has its limitations since it only takes into account a few genetic segments as compared to other techniques, such as whole-genome sequencing, which could be more informative in terms of comparing clonality on an international level. However, performing such analysis was not possible for this current study. The largest cluster of isolates (C6), mostly pertaining to IC II, caused a large outbreak in 2009 that persisted to 2013 (Table 1). This clone was detected in several hospital wards but most of the patients from whom the isolates were obtained have been admitted to the ICU and CCNU before being transferred to the other wards. Nevertheless, this reflects the reported success of IC II to persist in hospitals, whether on abiotic surfaces or in the patients’ gut flora [29], and cause repeated infections over prolonged periods of time. IC I and IC III were also detected in various clusters, but at lower incidences (Table 1). The prevalence of these three ICs is very similar to that reported by Villalón *et al*. [11] which investigated the dissemination of *A*. *baumannii* clones in Spain over 11 years. This shows a degree of uniformity in terms of distribution of these clones in the country.

A previous study demonstrated that clinical isolates, even those very successful at causing nosocomial outbreaks, have varying profiles of virulence [21]. This is in agreement with our results where the virulence profiles of individual isolates varied greatly. Nevertheless, some associations were detected that could help in predicting the degree of virulence of certain clones. Our data show an association between clones pertaining to ICs I and III, and attenuated virulence, as compared to those pertaining to IC II (p<0.005). This association could be useful for clinicians in this hospital in terms of adjusting treatment regimens based on the expected degree of virulence and the severity of the illness of the patient. Moreover, this information could be exploited by infection control specialists through the adaptation of eradication protocols to specific clones. Nevertheless, these associations were absent from the other study conducted by part of our research group using strains isolated from Lebanese patients [27]. The discrepancy in the results between the two sets of isolates suggests that the associations encountered in this current student are only local associations and cannot be globalized. Moreover, this further demonstrates the plasticity of *A*. *baumannii* [30] and the ability of clones to adapt differently in different environments. The different genetic environments in which the clones were present, and the exposure to different antimicrobial agents, could have resulted in the differential expression of virulence determinants in the different hospitals. Another important discrepancy between the two studies is that most of the isolates collected from Lebanese patients demonstrated surface motility [27], whereas most isolates in this study did not show surface motility. Also of note, although Difco agar was used, the isolates that did show diffusion patterns in both studies showed circular patterns, unlike what was reported for this type of agar [20]. This could suggest that the different types of patterns reported by Clemmer *et al*. [20] could be due to an experimental factor that is not restricted to the brand of the agar.

Most of the isolates (58.85%) in this study were collected in 2009. In general, fewer infections were caused by *A*. *baumannii* in subsequent years, indicating the implementation of good infection control protocols at this hospital. However, an alarmingly high rate of carbapenem non-sensitivity of 84.75% over 5 years was detected. This high rate is similar to that reported by Perez *et al*. [9], with the difference being the predominance of *bla*_OXA-24-like_ in our study, as opposed to the predominance of *bla*_OXA-23-like_ in the other [9]. Additionally, this rate is much higher than that reported for other European countries [10], revealing a worrisome situation in this country.

Even though *bla*_OXA-23-like_ is the most disseminated oxacillinase among *A*. *baumannii* isolates worldwide, *bla*_OXA-24-like_ seems to be more prevalent in Spain [16]. This was reflected in our study where *bla*_OXA-24-like_ was present in 62.71% of the isolates whereas *bla*_OXA-23-like_ was only detected in 11.86%. Harboring *bla*_OXA-24-like_ was associated with increased virulence as compared to harboring *bla*_OXA-23-like_ or *bla*_OXA-58-like_ (p<0.05). This association could be exploited in a similar manner to that of IC II in treatment regimens and infection control protocols in this Spanish hospital. When comparing the results of this study to the previous study performed on the Lebanese set of isolates, it became evident that the lack of *bla*_OXA-24-like_ in the previous study could have obscured statistical associations between virulence and clonality since this gene was only detected in two of the Lebanese isolates [27]. Furthermore, this suggests that the presence of this particular OXA, rather than clonality, is what is causing the association with increased virulence. Investigating larger pools of isolates harboring all three OXAs could help verify this hypothesis. Additionally, it is important to note that, since only exemplary sequence assessment was performed in this study, some associations between specific sub-types of the OXA families and the factors tested for might have been obscured.

A few isolates pertaining to different ICs were grouped into one pulsotype, probably due to the higher discriminatory power of PFGE. This is demonstrated in C6 where two isolates pertained to ICs I and III instead of IC II. This cluster also harbored two IC II isolates that were sensitive to carbapenems, which is a rare observation but one that has been previously reported [31]. This could be due to the loss of resistance resulting from the lack of antibiotic pressure over a period of time [32]. C6 also harbored one CRAB isolate with no carbapenemase gene detected, suggesting the acquisition of resistance through means that were not tested for in this study. Additionally, the intrinsic *bla*_OXA-51-like_ gene, that only results in carbapenem resistance if over-expressed [33], was missing from three isolates that were identified as *A*. *baumannii* by Matrix Assisted Laser Desorption Ionization—Time of Flight Mass Spectrometry (MALDI-TOF MS). This could suggest that these isolates harbor a variant of this gene that is not amplified by the primers that were used. However, a more possible explanation is that the databases used for the analysis of the MALDI-TOF MS results might have been outdated and did not have enough discriminatory power to differentiate between *A*. *baumannii* and other members of the *Acinetobacter calcoaceticus–Acinetobacter baumannii* complex that are harder to detect by this technique [34].

Most isolates in this study remain susceptible to amikacin and colistin. Susceptibility to the latter antibiotic is especially important due to the international trend to use colistin in the treatment of MDR *A*. *baumannii* infections [35]. Colistin resistance among *A*. *baumannii* has been previously reported in Spain [36]. Two colistin resistant isolates were encountered in our study. The first was isolate 12, which did not belong to any cluster nor IC, and the second was isolate 45, which was part of C6 and pertained to IC II. Interestingly, the former showed the slowest doubling time while the latter showed the fastest. Both showed α-hemolysis, although isolate 12 was slower in the development of hemolysis. Both were also non-motile. Isolate 12 harbored *bla*_OXA-24-like_ and did not produce siderophores, while isolate 45 harbored *bla*_OXA-58-like_, had a greater exoprotease production, and produced siderophores. Isolate 45 was only susceptible to amikacin and minocycline, whereas isolate 12 was susceptible to ampicillin/sulbactam, amikacin, netilmycin, and minocycline. Further genetic characterization of these isolates could help in understanding the different mechanisms in play that made isolate 45 highly virulent, highly resistant, and with a very fast doubling time. Additionally, it is worth mentioning that the isolate pertaining to Group 14 was sensitive to carbapenems, as opposed to its counterpart which was first detected in Romanian hospitals, was carbapenem-resistant, and harbored *bla*_OXA-58-like_ [37].

Doubling times are clearly affected by much more factors than are within the scope of this study, since they are affected by the entire metabolism of the cell. Therefore, doubling times were calculated for a few isolates with varying profiles, and when no pattern was detected, determination of doubling times for all the isolates was discontinued. Finally, it is worth mentioning that the highly susceptible sporadic isolate 57 showed the highest motility rate and proteolytic activity, produced strong biofilms, and was positive for siderophore production. Although the interplay between resistance and virulence seems to be a highly complex one, this observation could suggest that its lack of resistance could be attributing to its increased virulence. Performing a similar study on more sporadic isolates, and isolates from different origins and clonality could reveal further clinically important associations, and help better understand the interaction between antimicrobial resistance and virulence in *A*. *baumannii*.

## Materials and methods

### Bacterial strains

Fifty nine consecutive non-repetitive bloodstream isolates were collected from different wards of HU-LP, Madrid, Spain, from January 2009 until March 2013. The strains were not collected firsthand for the purposes of this study, but were rather collected during routine clinical procedures at Hospital Universitario—La Paz. They were stored as pure bacterial cultures in the collection of the Microbiology Department and this collection does not contain any human material. The strains were recovered from the collection and labelled for this study irreversibly dissociating them from the patient data. The study does not involve any human material or patient data, therefore no approval from the IRB was sought. Identification of the isolates was done using MALDI-TOF MS (Bruker Daltonik GmbH) as previously described [38]. The intrinsic *bla*_OXA-51-like_ gene was then amplified by PCR for confirmation [33]. The strains were stored in Luria-Bertani (LB) Broth with 20% glycerol at -20°C until used. *Pseudomonas fluorescens* strain B52 [39] was used as a positive control for surface motility, siderophore production, and determination of proteolytic activity. This bacterium was incubated at 30°C instead of 37°C whenever it was used.

### Antimicrobial susceptibility testing

MICs were determined using Vitek2 with AST-N-245 cards (BioMérieux Mercy l’Etoile, France). The antimicrobial agents used were: piperacillin (4–128 μg/mL), piperacillin/tazobactam (4/4-128/4 μg/mL), ticarcillin (4–128 μg/mL), ampicillin/sulbactam (2/2-32/16 μg/mL), cefepime (1–64 μg/mL), ceftazidime (1–64 μg/mL), meropenem (0.25–16 μg/mL), imipenem (0.25–16 μg/mL), colistin (0.5–16 μg/mL), amikacin (2–64 μg/mL), gentamicin (1–16 μg/mL), tobramycin (1–16 μg/mL), minocycline (1–16 μg/mL), levofloxacin (0.12–8 μg/mL), ciprofloxacin (0.25–4 μg/mL), and trimethoprim/sulfamethoxazole (1/19-16/304 μg/mL). E-tests (BioMérieux Mercy l’Etoile, France) were performed for verification of colistin resistance. The MIC cutoff values were implemented according to the CLSI [28] guidelines and the results were accordingly reported as resistant, intermediately resistant, or susceptible.

### Polymerase chain reactions

PCRs were performed for the detection of the *OmpA*, *CsuE*, *bla*_OXA-51-like_, *bla*_OXA-23-like_, *bla*_OXA-24-like_, *bla*_OXA-58-like_, *bla*_OXA-48_, *bla*_NDM_, and *bla*_KPC_ genes. The primers used, as well as the annealing temperatures for each primer pair, are found in S2 Table. Positive controls were obtained for each gene from the clinical microbiology laboratory of HU-LP. Amplicons were also randomly chosen and sent to Secugen S. L. (Madrid, Spain) for sequencing, in order to verify the amplicon sequences. The sequenced segments were uploaded to Genbank and given accession numbers ranging from KY514126 to KY514150, consecutively.

The thermocycling programs used consisted of an initial elongation at 94°C for 3 minutes; followed by 30 cycles of 94°C for 45 seconds, the respective annealing temperatures (S2 Table) for 45 seconds, and 72°C for 1 minute; and a final extension step at 72°C for 5 minutes. The number of cycles was increased to 36 and the annealing time was increased to 50 seconds for the *bla*_OXA-48_, *bla*_NDM_, and *bla*_KPC_ genes [40].

In order to determine the global lineage of the strains, two multiplex PCRs proposed by Turton *et al*. [41] that target specific alleles of the *ompA*, *csuE*, and *bla*_OXA-51-like_ genes were performed. The primers for each Multiplex PCR are listed in S3 Table. Using a classification scheme summarized by Karah *et al*. [8], the strains were classified as belonging to one out of 15 identified groups based on the combination of the different alleles amplified. Group 1 of this scheme corresponds to IC II, Group 2 to IC I, and Group 3 to IC III [8].

### Pulsed field gel electrophoresis

PFGE was performed using the ApaI restriction enzyme as previously described [42]. Restriction fragments were separated in 1% agarose gels using the Contour-Clamped Homogeneous Electric Field (CHEF) apparatus (Bio-Rad, Munich, Germany). The visualized fragments were analyzed using the Bionumerix software version 6.6 (Applied Maths, St-Martens-Latem, Belgium) and the dendrogram was constructed using the Unweighted Pair Group Method with Arithmetic Mean (UPGMA) with 1.5% tolerance and 1.5% optimization. 80% similarity was considered as the cutoff value for considering the generated pattern as pertaining to the same cluster.

### Surface motility

In order to detect surface motility, a single colony was grown overnight in LB at 37°C. The following day, the turbidity of the suspension was adjust to be equivalent to 0.5McFarland (around 10^8^ CFU/mL) and 1µL was plated on 0.3% LB-Agar (Difco, BD, USA) plates. The plates were incubated at 37°C for 14 hours and the diameter of the motility diffusion pattern was measured [20]. This, and all subsequent experiments were performed in independent triplicates.

### Biofilm formation

In order to detect biofilms, inoculation of 1mL LB broth in polystyrene tubes with one isolated colony was performed. The tubes were then incubated overnight at 37°C, stained with 1% crystal violet for 10 minutes, washed with water, and left to dry [19]. Strong ring formations, indicated by a heavy colorization at the liquid-air interface, were reported as “++”. Weak ring formations, indicated by a very thin colorized ring, were reported as “+”. The absence of any colorization in the test tube was recorded as “-”.

### Hemolytic activity

In order to detect hemolytic activity, LB broth was inoculated with a single colony from the tested strain and incubated at 37°C overnight. The suspension was then adjusted to 10^6^ CFU/mL and 10µL were plated on 5% Sheep Blood Agar (SBA) (Biomérieux Mercy L’Etoile, France). The plates were incubated at 37°C and observed for 6 days [43].

### Proteolytic activity

Proteolytic activity of the tested strains was determined as previously described [21]. One colony was incubated overnight in Trypticase Soy Broth Dialysate overnight at 37°C with shaking at 200 rpm. The bacterial suspension was then centrifuged at 4000×g for 10 minutes and the supernatant was filter sterilized. 500µL of 1mg/mL Azoalbumin solution dissolved in 50mM Tris-HCl (pH = 7.7) was then incubated with 500µL of the supernatant at 37°C for 24 hours with gentle shaking. Then, trichloroacetic acid was added to each tube with a final concentration of 13% and the tubes were incubated at -20°C for 20 minutes. This was followed by centrifugation at 15,000×g for 10 minutes and measurement of the OD_440_ of the supernatant. U/L values were calculated as previously described [44] where one U was defined as the amount of enzyme needed to degrade one micromole of Azoalbumin.

### Siderophore production

For the determination of siderophore production, one colony was used to inoculate 5mL of the iron-deprived PMS_7_-Ca medium. The suspension was then incubated for 72 hours with shaking at 200 rpm. Then, the suspensions were centrifuged at 4000×g for 10 minutes and the supernatant was filter sterilized. The Chrome Azurol Solution (CAS) was also prepared as described by Louden *et al*. [45]. 1 mL of this solution was incubated with 1 mL of the filter sterilized supernatant for one hour and the OD_630_ was measured. Un-inoculated PMS_7_-Ca and CAS were used as a reference measurement. A 10% difference between the sample and the reference was considered as positive [46].

### Growth curves

Doubling times were calculated for representative strains from each cluster with varying susceptibility profiles. 500µL from bacterial cultures grown overnight at 37°C in LB broth were diluted in 50mL fresh LB broth. The suspension was then incubated at 37°C with shaking at 200 rpm for 8 hours and the OD_600_ was measured, in duplicate, each hour (Hitachi, U-1900 Spectrophotometer, Tokyo, Japan). Doubling times were calculated from two measurements that fell within the exponential phase [47].

### Statistical analysis

All statistical analyses were performed using the SPSS program, version 17.0 (SPSS 111 Inc., Chicago, USA). The chi squared test and Fisher’s exact test (two sided) were used in order to analyze the qualitative data. Normal distribution of the quantitative data was tested for using the Shapiro-Wilk test. One-way ANOVA and the student t-tests were performed for the comparison of normally distributed data, while the Kruskal-Wallis and the Mann-Whitney tests were used for the comparison of non-normally distributed data. P values of less than 0.05 were considered as statistically significant for all the statistical tests performed.

## Conclusions

In conclusion, a very high level of carbapenem non-susceptibility in a Spanish hospital was detected over a period of five years. IC II and *bla*_OXA-24-like_ were predominant in this hospital, although other ICs and OXAs were detected. IC II and *bla*_OXA-24-like_ were also associated with increased virulence as compared to the other ICs and OXAs. This association could be locally exploited by clinicians and infection control specialists in order to improve on patient outcome, especially since determination of clonality and presence of *bla*_OXA-24-like_ could be performed rapidly. Future investigations using larger and more diversified pools of isolates could shed further light on these associations and help better understand the complex mechanisms in play that affect virulence and antibiotic resistance.

## Supporting information

S1 Table Minimum Inhibitory Concentrations (MICs) of the tested antimicrobial agents along with the interpretation according to CLSI guidelines. “R” stands for Resistant, “I” stands for Intermediately Resistant, and “S” stands for susceptible. “TIC” stands for ticarcillin, “PIP” for piperacillin, “A/S” for ampicillin/sulbactam, “P/T” for piperacillin/tazobactam, “CTZ” for ceftazidime, “CFP” for cefepime, “IMI” for imipenem, “MER” for meropenem, “COL” for colistin, “G” for gentamicin, “TO” for tobramycin, “AK” for amikacin, “MIN” for minocycline, “CIP” for ciprofloxacin, “LEV” levofloxacin, and “T/S” for trimethoprim/sulfamethoxazole.
(DOCX)

S2 Table Primers used with their respective annealing temperatures. (DOCX)

S3 Table Primers used in the identification of the global lineages of the strains. (DOCX)
