# Supplementary material for: Clonality, virulence determinants, and profiles of resistance of clinical Acinetobacter baumannii isolates obtained from a Spanish hospital
Source: PLoS One. 2017 Apr 27;12(4):e0176824. doi: 10.1371/journal.pone.0176824 (PMC5407824; doi:10.1371/journal.pone.0176824)
Supplement: S1 Table — “R” stands for Resistant, “I” stands for Intermediately Resistant, and “S” stands for susceptible. “TIC” stands for ticarcillin, “PIP” for piperacillin, “A/S” for ampicillin/sulbactam, “P/T” for piperacillin/tazobactam, “CTZ” for ceftazidime, “CFP” for cefepime, “IMI” for imipenem, “MER” for meropenem, “COL” for colistin, “G” for gentamicin, “TO” for tobramycin, “AK” for amikacin, “MIN” for minocycline, “CIP” for ciprofloxacin, “LEV” levofloxacin, and “T/S” for trimethoprim/sulfamethoxazole. (DOCX) [file pone.0176824.s001.docx]

**S1 Table. Minimum Inhibitory Concentrations (MICs) of the tested antimicrobial agents along with the interpretation according to CLSI guidelines.** “R” stands for Resistant, “I” stands for Intermediately Resistant, and “S” stands for susceptible. “TIC” stands for ticarcillin, “PIP” for piperacillin, “A/S” for ampicillin/sulbactam, “P/T” for piperacillin/tazobactam, “CTZ” for ceftazidime, “CFP” for cefepime, “IMI” for imipenem, “MER” for meropenem, “COL” for colistin, “G” for gentamicin, “TO” for tobramycin, “AK” for amikacin, “MIN” for minocycline, “CIP” for ciprofloxacin, “LEV” levofloxacin, and “T/S” for trimethoprim/sulfamethoxazole.

| **Strain** | **TIC** | **PIP** | **A/S** | **P/T** | **CTZ** | **CFP** | **IMI** | **MER** | **COL** | **G** | **TO** | **AK** | **MIN** | **CIP** | **LEV** | **T/S** |
| --- | --- | --- | --- | --- | --- | --- | --- | --- | --- | --- | --- | --- | --- | --- | --- | --- |
| 1 | > 64 | > 64 | >16/8 | > 64/4 | >32 | >32 | >8 | >8 | ≤0.5 | 4 | ≤1 | ≤2 | ≤1 | >2 | >4 | >4/76 |
| 3 | > 64 | > 64 | 16 | > 64/4 | >32 | 16 | >8 | >8 | ≤0.5 | ≤1 | ≤1 | ≤2 | 2 | >2 | >4 | >4/76 |
| 4 | > 64 | > 64 | >16/8 | > 64/4 | >32 | >32 | >8 | >8 | ≤0.5 | 8 | ≤1 | ≤2 | 2 | >2 | >4 | >4/76 |
| 5 | > 64 | > 64 | 16 | > 64/4 | >32 | >32 | >8 | >8 | ≤0.5 | >8 | >8 | >32 | 8 | >2 | >4 | >4/76 |
| 6 | > 64 | > 64 | >16/8 | > 64/4 | >32 | >32 | >8 | >8 | ≤0.5 | 8 | ≤1 | ≤2 | 2 | >2 | >4 | >4/76 |
| 7 | > 64 | > 64 | 16 | > 64/4 | >32 | >32 | >8 | >8 | 2 | >8 | >8 | >32 | 8 | >2 | >4 | >4/76 |
| 8 | > 64 | > 64 | >16/8 | > 64/4 | >32 | >32 | >8 | >8 | ≤0.5 | >8 | >8 | >32 | 8 | >2 | >4 | >4/76 |
| 9 | > 64 | > 64 | 16 | > 64/4 | >32 | 32 | >8 | >8 | 2 | >8 | >8 | >32 | 8 | >2 | >4 | >4/76 |
| 10 | > 64 | > 64 | >16/8 | > 64/4 | >32 | >32 | >8 | >8 | 2 | >8 | >8 | >32 | 8 | >2 | >4 | >4/76 |
| 11 | > 64 | > 64 | 16 | > 64/4 | >32 | >32 | >8 | >8 | ≤0.5 | >8 | >8 | >32 | 8 | >2 | >4 | >4/76 |
| 12 | 64 | > 64 | 4 | > 64/4 | 16 | 32 | >8 | >8 | >8 | >8 | >8 | 8 | ≤1 | >2 | >4 | >4/76 |
| 13 | > 64 | > 64 | 16 | > 64/4 | >32 | >32 | >8 | >8 | ≤0.5 | >8 | >8 | 16 | ≤1 | >2 | >4 | >4/76 |
| 14 | > 64 | > 64 | 16 | > 64/4 | >32 | >32 | >8 | >8 | ≤0.5 | >8 | >8 | 16 | ≤1 | >2 | >4 | >4/76 |
| 15 | > 64 | > 64 | 16 | > 64/4 | >32 | >32 | >8 | >8 | ≤0.5 | >8 | >8 | 16 | ≤1 | >2 | >4 | >4/76 |
| 16 | 32 | 16 | ≤2/1 | 8 | 16 | 2 | ≤1 | 1 | ≤0.5 | ≤1 | ≤1 | ≤2 | ≤1 | >2 | 1 | ≤2/38 |
| 17 | > 64 | > 64 | 16 | > 64/4 | 32 | >32 | >8 | >8 | ≤0.5 | >8 | >8 | >32 | ≤1 | >2 | >4 | >4/76 |
| 20 | > 64 | > 64 | 16 | > 64/4 | >32 | >32 | >8 | >8 | ≤0.5 | >8 | >8 | >32 | 8 | >2 | >4 | >4/76 |
| 21 | > 64 | > 64 | >16/8 | > 64/4 | >32 | >32 | >8 | >8 | ≤0.5 | >8 | 2 | >32 | >8 | >2 | >4 | >4/76 |
| 22 | > 64 | > 64 | >16/8 | > 64/4 | >32 | >32 | >8 | >8 | ≤0.5 | >8 | 2 | >32 | >8 | >2 | >4 | >4/76 |
| 23 | > 64 | > 64 | >16/8 | > 64/4 | >32 | >32 | >8 | >8 | ≤0.5 | >8 | >8 | >32 | >8 | >2 | >4 | >4/76 |
| 24 | > 64 | > 64 | >16/8 | > 64/4 | >32 | >32 | >8 | >8 | ≤0.5 | 4 | ≤1 | ≤2 | ≤1 | >2 | >4 | >4/76 |
| 25 | > 64 | > 64 | >16/8 | > 64/4 | >32 | >32 | >8 | >8 | ≤0.5 | 4 | ≤1 | ≤2 | ≤1 | >2 | >4 | >4/76 |
| 26 | 32 | > 64 | 4 | > 64/4 | >32 | 16 | ≤1 | 1 | 1 | ≤1 | ≤1 | ≤2 | 2 | >2 | >4 | >4/76 |
| 27 | > 64 | > 64 | >16/8 | > 64/4 | >32 | >32 | >8 | >8 | ≤0.5 | >8 | ≤1 | ≤2 | ≤1 | >2 | >4 | >4/76 |
| 28 | > 64 | > 64 | >16/8 | > 64/4 | >32 | >32 | >8 | >8 | ≤0.5 | 2 | ≤1 | ≤2 | ≤1 | >2 | >4 | >4/76 |
| 29 | > 64 | > 64 | >16/8 | > 64/4 | >32 | >32 | >8 | >8 | ≤0.5 | 4 | ≤1 | ≤2 | ≤1 | >2 | >4 | >4/76 |
| 30 | > 64 | > 64 | 4 | 64 | 16 | 16 | 2 | 2 | ≤0.5 | >8 | 8 | 4 | ≤1 | >2 | >4 | >4/76 |
| 31 | 16 | 16 | 4 | 16 | 4 | 2 | ≤1 | 1 | ≤0.5 | ≤1 | ≤1 | ≤2 | ≤1 | ≤0.25 | ≤0.12 | ≤2/38 |
| 32 | ≤8 | 16 | ≤2/1 | ≤4/4 | 4 | 2 | ≤1 | ≤0.25 | ≤0.5 | ≤1 | ≤1 | ≤2 | ≤1 | ≤0.25 | ≤0.12 | ≤2/38 |
| 33 | > 64 | > 64 | >16/8 | > 64/4 | >32 | >32 | >8 | >8 | ≤0.5 | 8 | ≤1 | ≤2 | ≤1 | >2 | >4 | >4/76 |
| 34 | > 64 | > 64 | >16/8 | > 64/4 | 32 | 16 | >8 | >8 | ≤0.5 | >8 | >8 | ≤2 | ≤1 | >2 | >4 | >4/76 |
| 35 | > 64 | > 64 | >16/8 | > 64/4 | 32 | 8 | >8 | 8 | ≤0.5 | >8 | >8 | ≤2 | ≤1 | >2 | >4 | >4/76 |
| 36 | > 64 | > 64 | >16/8 | > 64/4 | 32 | 16 | >8 | 8 | ≤0.5 | >8 | >8 | ≤2 | ≤1 | >2 | >4 | >4/76 |
| 37 | > 64 | > 64 | >16/8 | > 64/4 | 32 | 8 | >8 | 8 | ≤0.5 | >8 | >8 | ≤2 | ≤1 | >2 | >4 | >4/76 |
| 38 | ≤8 | 8 | ≤2/1 | ≤4/4 | 4 | 2 | ≤1 | ≤0.25 | ≤0.5 | ≤1 | ≤1 | ≤2 | ≤1 | ≤0.25 | ≤0.12 | ≤2/38 |
| 39 | > 64 | > 64 | >16/8 | > 64/4 | >32 | >32 | >8 | >8 | ≤0.5 | 4 | ≤1 | ≤2 | ≤1 | >2 | >4 | >4/76 |
| 40 | > 64 | > 64 | >16/8 | > 64/4 | >32 | >32 | >8 | >8 | ≤0.5 | 4 | ≤1 | ≤2 | ≤1 | >2 | >4 | >4/76 |
| 41 | > 64 | > 64 | >16/8 | > 64/4 | 32 | 8 | >8 | >8 | ≤0.5 | >8 | >8 | ≤2 | ≤1 | >2 | >4 | >4/76 |
| 42 | > 64 | > 64 | >16/8 | > 64/4 | 32 | 8 | >8 | >8 | ≤0.5 | >8 | >8 | ≤2 | ≤1 | >2 | >4 | >4/76 |
| 43 | > 64 | > 64 | >16/8 | > 64/4 | >32 | >32 | >8 | >8 | ≤0.5 | 4 | ≤1 | ≤2 | ≤1 | >2 | >4 | >4/76 |
| 44 | > 64 | > 64 | >16/8 | > 64/4 | >32 | >32 | >8 | >8 | ≤0.5 | 4 | ≤1 | ≤2 | ≤1 | >2 | >4 | >4/76 |
| 45 | > 64 | > 64 | >16/8 | > 64/4 | 16 | 8 | >8 | 4 | >8 | >8 | 8 | ≤2 | 2 | >2 | >4 | >4/76 |
| 46 | 16 | 16 | 4 | ≤4/4 | 4 | 2 | ≤1 | ≤0.25 | ≤0.5 | ≤1 | ≤1 | ≤2 | ≤1 | ≤0.25 | ≤0.12 | ≤2/38 |
| 47 | > 64 | > 64 | 16 | > 64/4 | >32 | 16 | >8 | >8 | ≤0.5 | ≤1 | ≤1 | ≤2 | 2 | >2 | >4 | >4/76 |
| 48 | > 64 | > 64 | >16/8 | > 64/4 | 16 | 32 | >8 | >8 | 2 | 8 | ≤1 | ≤2 | ≤1 | >2 | >4 | >4/76 |
| 49 | > 64 | > 64 | >16/8 | > 64/4 | >32 | >32 | >8 | >8 | ≤0.5 | 8 | ≤1 | ≤2 | ≤1 | >2 | >4 | >4/76 |
| 50 | > 64 | > 64 | >16/8 | > 64/4 | >32 | >32 | >8 | >8 | ≤0.5 | 2 | ≤1 | ≤2 | ≤1 | >2 | >4 | >4/76 |
| 51 | > 64 | > 64 | >16/8 | > 64/4 | >32 | >32 | >8 | >8 | ≤0.5 | 4 | ≤1 | ≤2 | ≤1 | >2 | >4 | >4/76 |
| 52 | > 64 | > 64 | >16/8 | > 64/4 | >32 | >32 | >8 | >8 | ≤0.5 | 4 | ≤1 | ≤2 | ≤1 | >2 | >4 | >4/76 |
| 53 | > 64 | > 64 | 4 | > 64/4 | 16 | 16 | 8 | 8 | ≤0.5 | >8 | >8 | 16 | ≤1 | >2 | >4 | >4/76 |
| 54 | > 64 | > 64 | >16/8 | > 64/4 | >32 | >32 | >8 | >8 | ≤0.5 | 4 | ≤1 | ≤2 | ≤1 | >2 | >4 | >4/76 |
| 55 | > 64 | > 64 | >16/8 | > 64/4 | >32 | >32 | >8 | >8 | ≤0.5 | 4 | ≤1 | ≤2 | ≤1 | >2 | >4 | >4/76 |
| 56 | > 64 | > 64 | >16/8 | > 64/4 | >32 | >32 | >8 | >8 | ≤0.5 | >8 | 8 | 8 | ≤1 | >2 | >4 | >4/76 |
| 57 | 8 | 16 | ≤2/1 | ≤4/4 | 4 | 2 | ≤1 | ≤0.25 | ≤0.5 | ≤1 | ≤1 | ≤2 | ≤1 | ≤0.25 | ≤0.12 | ≤2/38 |
| 58 | > 64 | > 64 | >16/8 | > 64/4 | >32 | >32 | >8 | >8 | ≤0.5 | >8 | 8 | 8 | ≤1 | >2 | >4 | >4/76 |
| 59 | ≤8 | 8 | ≤2/1 | ≤4/4 | 2 | 2 | ≤1 | ≤0.25 | ≤0.5 | ≤1 | ≤1 | ≤2 | ≤1 | ≤0.25 | ≤0.12 | ≤2/38 |
| 60 | > 64 | > 64 | >16/8 | > 64/4 | >32 | >32 | >8 | >8 | ≤0.5 | 4 | ≤1 | ≤2 | ≤1 | >2 | >4 | >4/76 |
| 61 | > 64 | > 64 | >16/8 | > 64/4 | >32 | >32 | >8 | >8 | ≤0.5 | 4 | ≤1 | ≤2 | ≤1 | >2 | >4 | >4/76 |
| 62 | > 64 | > 64 | >16/8 | > 64/4 | >32 | >32 | >8 | >8 | ≤0.5 | 4 | ≤1 | ≤2 | ≤1 | >2 | >4 | >4/76 |
