# Supplementary material for: Clonality, virulence determinants, and profiles of resistance of clinical Acinetobacter baumannii isolates obtained from a Spanish hospital
Source: PLoS One. 2017 Apr 27;12(4):e0176824. doi: 10.1371/journal.pone.0176824 (PMC5407824; doi:10.1371/journal.pone.0176824)
Supplement: S2 Table — (DOCX) [file pone.0176824.s002.docx]

**S2 Table.** **Primers used with their respective annealing temperatures.**

| **Primer** | **Sequence** | **Annealing Temperature** | **Reference** |
| --- | --- | --- | --- |
| OmpA-F | 5’-CAATTGTTATCTCTGGAG-3’ | 50˚C | [1] |
| OmpA-R | 5’-ACCTTGAGTAGACAAACGA-3’ |  |  |
| CsuE-F | 5’-ATGCATGTTCTCTGGACTGATGTTGAC-3’ | 65˚C | [1] |
| CsuE-R | 5’-CGACTTGTACCGTGACCGTATCTTGATAAG-3’ |  |  |
| Oxa-51-like-F | 5’-ATGAACATTAAAGCACTC-3’ | 46˚C | [1] |
| Oxa-51-like-R | 5’-CTATAAAATACCTAATTGTTC-3’ |  |  |
| Oxa-23-like-F | 5’-GATCGGATTGGAGAACCAGA-3’ | 53˚C | [2] |
| Oxa-23-like-R | 5’-ATTTCTGACCGCATTTCCAT-3’ |  |  |
| Oxa-24-like-F | 5’-GGTTAGTTGGCCCCCTTAAA-3’ | 53˚C | [2] |
| Oxa-24-like-R | 5’-AGTTGAGCGAAAAGGGGATT-3’ |  |  |
| Oxa-58-like-F | 5’-AAGTATTGGGGCTTGTGCTG-3’ | 53˚C | [2] |
| Oxa-58-like-R | 5’-CCCCTCTGCGCTCTACATAC-3’ |  |  |
| KPC-F | 5’-CGTCTAGTTCTGCTGTCTTG-3’ | 52˚C | [3] |
| KPC-R | 5’-CTTGTCATCCTTGTTAGGCG-3’ |  |  |
| NDM-F | 5’-GGTTTGGCGATCTGGTTTTC-3’ | 52˚C | [3] |
| NDM-R | 5’-CGGAATGGCTCATCACGATC-3’ |  |  |
| Oxa-48-F | 5’-GCGTGGTTAAGGATGAACAC-3’ | 52˚C | [3] |
| Oxa-48-R | 5’-CATCAAGTTCAACCCAACCG-3’ |  |  |

**References:**

[1] Turton JF, Woodford N, Glover J, Yarde S, Kaufmann ME, Pitt TL. Identification of *Acinetobacter baumannii* by detection of the blaOXA-51-like carbapenemase gene intrinsic to this species. Journal of Clinical Microbiology. 2006;44: 2974-2976.

[2] Mostachio AK, van der Heidjen I, Rossi F, Levin AS, Costa SF. Multiplex PCR for rapid detection of genes encoding oxacillinases and metallo β-lactamases in carpabenem-resistant *Acinetobacter* spp. Journal of Medical Microbiology. 2009;58: 1522-1524.

[3] Poirel L, Walsh TR, Cuviller V, Nordmann P. Multiplex PCR for detection of acquired carbapenemase genes. Diagnostic Microbiology and Infectious Disease. 2011;70: 119-123.
