# Supplementary material for: Clonality, virulence determinants, and profiles of resistance of clinical Acinetobacter baumannii isolates obtained from a Spanish hospital
Source: PLoS One. 2017 Apr 27;12(4):e0176824. doi: 10.1371/journal.pone.0176824 (PMC5407824; doi:10.1371/journal.pone.0176824)
Supplement: S3 Table — (DOCX) [file pone.0176824.s003.docx]

**S3 Table.** **Primers used in the identification of the global lineages of the strains.**

| **Multiplex 1** | | |
| --- | --- | --- |
| **Primer** | **Sequence** | **Amplicon Size (bp)** |
| Group1ompAF306 | 5’-GATGGCGTAAATCGTGGTA-3’ | 355 |
| Group1and2ompAR660 | 5’-CAACTTTAGCGATTTCTGG-3’ |  |
| Group1csuEF | 5’-CTTTAGCAAACATGACCTACC-3’ | 702 |
| Group1csuER | 5’-TACACCCGGGTTAATCGT-3’ |  |
| Gp1OXA66F89 | 5’-GCGCTTCAAAATCTGATGTA-3’ | 559 |
| Gp1OXA66R647 | 5’-GCGTATATTTTGTTTCCATTC-3’ |  |
| **Multiplex 2** | | |
| Group2ompAF378 | 5’-GACCTTTCTTATCACAACGA-3’ | 343 |
| Group1and2ompAR660 | 5’-CAACTTTAGCGATTTCTGG-3’ |  |
| Group2csuEF | 5’-GGCGAACATGACCTATTT-3’ | 580 |
| Group2csuER | 5’-CTTCATGGCTCGTTGGTT-3’ |  |
| Gp2OXA69F169 | 5’-CATCAAGGTCAAACTCAA-3’ | 162 |
| Gp2OXA69R330 | 5’-TAGCCTTTTTTCCCCATC-3’ |  |
